# Dr. Frank L. Rea

**Published:** 1883-12

**Authors:** 


Dr. Frank L. Rea died August 10, 1883, of facial carbuncle, aged 24 years. The disease appeared July 31 as an inflamed hair follicle, causing sensitiveness. The hair was extracted by a friend, and found to be bulbless. It seemed to make an instantaneous and fatal impression on him, and he made an ominous record of the fact in his diary. He wrote :

July 30. A little boil is progressing rapidly on my upper lip to-day.

July 31. Have a boil or carbuncle on my upper lip. *Wonder if it will turn out as Egle's did last summer—i. e., kill me.* Professor prescribes 10 drops Fowler's solution 4 times a day. The carbuncle started yesterday morning as a little sore pimple, and I had Jacobson look at it, and from its apex drew a loose hair, from which the bulb was absent. I then had him paint it with carbolic acid. To-day it has progressed—not being very painful. P. M.—More painful ; painted it with atropine, relieving the pain also. Made an incision into it and introducing carbolic acid. It has swollen half way up to my eyelid, and is bearing in that direction. Pulse 96, temperature 99.2 at 9 P. M. I am fully aware of its dangers—Holmes' Surgery, page 890 ; Bryant, 140. *No post mortem, please.* The surface looks red and angry. Beverly Egle's case ran along as mine for a number of days, probably ten, after it commenced dying of suppurative phlebitis, extending up to the orbit and along the ophthalmic vein into the cavernous sinus. The island of Reil was a mass of pus, and infarctions were found in the lungs.

August 1. Lip and face markedly more swollen this morning, but not painful. The redness is confined mostly to the surface of the boil. Was out on business this forenoon, and this afternoon attended Professor's clinic at the College, and went over to County Hospital to witness an ovariotomy by Prof. Fenger, but did not feel well enough to stay.

Temperature 92.2 at noon, and 9 P. M., 99.6; pulse 108. Began tincture opii. 15 drops with each dose of Fowler, and oftener if pain requires it.

August 2. Taking on a distinctly carbuncular character. Brawny swelling with tubercular elevations. Pulse 96, temperature 100.

At 2 P. M. took ether, and Professor made an incision across through the indurated tissues about two inches long, and then cauterized thoroughly with caustic potass. the involved tissues. No pus found except size of a pea at the seat of original pimple.

Linseed poultice applied, and took $\frac{1}{4}$ gr. morphine. Not much pain this afternoon. Ether produced severe vesical irritation. Took three compound cathartic pills at bed time.

August 3. Lip and face very painful this morning. Pills operated well. Pulse 96, temperature 99.6. It has crossed the median line to the other half of lip. Professor injected 15 m. of 1-40 carbolic acid, and painted the surface with pure liquid carbolic acid. P. M.—Pulse 108, temperature 101$\frac{3}{4}$. No chill but chilly sensations. Began bromides; stopped poultice and use carbolic acid lotion.

This ended his personal record of his case, and is taken verbatim from his diary.

August 4. Symptom growing worse, fever high; shiny hot face very much swollen. Pulse 120, temperature 102. Opened left side of lip and cauterized it as the other.

August 5. Face excessively painful; swelling increases; œdematous condition of sides of face from obstruction of veins. Pain across chest, which settled into a severe pleuritic pain in right side. No cough or expectoration. This pain disappeared in a day or two under warm applications. Pulse 120 to 130, temperature 103$\frac{1}{4}$.

Sulph. quin 20 grains every 12 hours; mu. tincture ferri 40 drops every 4 hours; omit Fowler's solution.

August 6. Met with Dr. John E. Owens in consultation. A red band extended up left side of face an inch wide, reaching from lip to orbit. Œdema still continues increasing—120, 104½. Delusions begin to manifest themselves. No change of treatment. Opened further, and cauterized the left wound.

August 7. Mind markedly disturbed. No other marked change. Temperature, A. M., 103¼; P. M., 103¾. Pulse, 120 A. M; 108-120 P. M. Iodoform applied to wounds.

August 8. Delirium increasing,—describes what he sees as an infinite number of suicides on the walls and ceilings of his room. Then a change in which the whole scene is a broad, quiet ocean. Can only be arrested in his wanderings for a few minutes a time; pulse and temperature rising; lip swollen intensely; openings appear on the mucus surface of the lip; complains of pain across the forehead, and has for some days. Pleuritic pain ceased.

August 9. Very delirious all night; symptoms all worse; had to be restrained till near morning, at which time he became unconscious; so remained till he died—10th at 11 A. M.

There is probably little reason to doubt the pathology, as the disease is described and here confirmed as suppurative phelebetis, and the anatomical connection between the lip and brain promise the best explanation of its exceptionally fatal tendency. There was a strumous tendency in the case, but whether it modified it is uncertain. But little need be added. The marked peculiarities were its uninterrupted progress, and his unmoved opinion of its necessary fatality. I do not think he ever wavered for a moment on this, and tho', when I reasoned with him and endeavored to divert his mind and show him the direct tendency of this central fact in attracting the disease and producing the very thing we were trying to avert, he would seem to assent to me, and he would announce the fatal prospect to the next person to whom he talked.

He predicted the progress of the disease, the occurrence and time of his delirium, and arranged his affairs with a quiet confidence born of conviction, and no amount of ridicule or reason affected him in the least; and this was not the result of superstition, but of knowledge and experience and a distrust of his recuperative power, alarmed as he always was at disease in himself.

In a similar case I should feel at a loss for any suitable treatment, but if the nature of the disease is properly diagnosed I should be tempted to destroy the facial and cerebral venous connection early by a transverse incision extending from the side of the nose across beneath the orbits, cauterizing it with actual cautery, caustic, potash or strong acid, following this with the applications to the region so treated with proper constitutional treatment.

Dr. Rea was a young man of unusual promise, a careful, diligent student, kind disposition, winning manners, and bade fair to attain an enviable position in his profession. He graduated at the Chicago Medical College in 1882, and was appointed interne to St. Luke's, completing his term of service April last. His loss will be deeply felt in the positions he held, the College of Physicians and Surgeons, as well as by his numerous friends and acquaintances, but more than all by his heartbroken parents whose bright hopes and fond anticipations for him were doomed to such sudden blight. R. L. R.

